# Exploring the Effects of In-App Components on Engagement With a Symptom-Tracking Platform Among Participants With Major Depressive Disorder (RADAR-Engage): Protocol for a 2-Armed Randomized Controlled Trial

**DOI:** 10.2196/32653

**Published:** 2021-12-21

**Authors:** Katie M White, Faith Matcham, Daniel Leightley, Ewan Carr, Pauline Conde, Erin Dawe-Lane, Yatharth Ranjan, Sara Simblett, Claire Henderson, Matthew Hotopf

**Affiliations:** 1 Department of Psychological Medicine Institute of Psychiatry, Psychology & Neuroscience King’s College London London United Kingdom; 2 Department of Biostatistics and Health Informatics Institute of Psychiatry, Psychology & Neuroscience King's College London London United Kingdom; 3 Department of Psychology Institute of Psychiatry, Psychology & Neuroscience King's College London London United Kingdom; 4 Health Service & Population Research Department Institute of Psychiatry, Psychology & Neuroscience King's College London London United Kingdom; 5 South London and Maudsley National Health Service Foundation Trust London United Kingdom

**Keywords:** app, engagement, major depressive disorder, remote measurement technologies, research, mobile phone

## Abstract

**Background:**

Multi-parametric remote measurement technologies (RMTs) comprise smartphone apps and wearable devices for both active and passive symptom tracking. They hold potential for understanding current depression status and predicting future depression status. However, the promise of using RMTs for relapse prediction is heavily dependent on user engagement, which is defined as both a behavioral and experiential construct. A better understanding of how to promote engagement in RMT research through various in-app components will aid in providing scalable solutions for future remote research, higher quality results, and applications for implementation in clinical practice.

**Objective:**

The aim of this study is to provide the rationale and protocol for a 2-armed randomized controlled trial to investigate the effect of insightful notifications, progress visualization, and researcher contact details on behavioral and experiential engagement with a multi-parametric mobile health data collection platform, Remote Assessment of Disease and Relapse (RADAR)–base.

**Methods:**

We aim to recruit 140 participants upon completion of their participation in the RADAR Major Depressive Disorder study in the London site. Data will be collected using 3 weekly tasks through an active smartphone app, a passive (background) data collection app, and a Fitbit device. Participants will be randomly allocated at a 1:1 ratio to receive either an adapted version of the active app that incorporates insightful notifications, progress visualization, and access to researcher contact details or the active app as usual. Statistical tests will be used to assess the hypotheses that participants using the adapted app will complete a higher percentage of weekly tasks (behavioral engagement: primary outcome) and score higher on self-awareness measures (experiential engagement).

**Results:**

Recruitment commenced in April 2021. Data collection was completed in September 2021. The results of this study will be communicated via publication in 2022.

**Conclusions:**

This study aims to understand how best to promote engagement with RMTs in depression research. The findings will help determine the most effective techniques for implementation in both future rounds of the RADAR Major Depressive Disorder study and, in the long term, clinical practice.

**Trial Registration:**

ClinicalTrials.gov NCT04972474; http://clinicaltrials.gov/ct2/show/NCT04972474

**International Registered Report Identifier (IRRID):**

DERR1-10.2196/32653

## Introduction

### Background

The last decade has seen a significant increase in the use of mobile technology in health care (mobile health [mHealth]) research and clinical practice [1]. One such application of mHealth is the use of remote measurement technologies (RMTs), which provide real-time, longitudinal health tracking using a combination of smartphone apps for active symptom reporting tasks (active RMT [aRMT]) and mobile or wearable sensors for passive data collection (passive RMT [pRMT]) [2]. Multi-parametric RMT data have the potential to inform about current clinical state by reflecting patients’ daily experiences in situ. They may also offer predictions by detecting subtle shifts in physiological, behavioral, or environmental variables that occur before a change in clinical state [3,4].

RMTs may be particularly relevant in recurrent conditions. Major depressive disorder (MDD) is a mental health disorder characterized by persistent low mood and anhedonia, often following a trajectory of remission and relapse over time [5]. The economic burden of MDD is currently estimated at US $326 billion [6], with increased risks of comorbidities and health care use associated with high relapse rates [7]. RMTs can collect information about a wide range of factors associated with MDD (mood variability, sociability, activity, cognition, and sleep) [2]. Raw, passive sensor data can be translated into low-level features, higher-level behavioral markers, and ultimately clinical state [8]. Previous work has found ambulatory self-reporting of mood symptoms [9] and multi-parametric RMT measures of location, device use, and sleep across a 30-day period [10] to be clinically valid assessments of individual depression trajectories.

The benefits of using RMTs for MDD symptom tracking are 2-fold. First, given the suggested biases [11] toward mood-congruent information in symptom reporting in depression, such data present a more accurate picture of symptom variability. Second, continuous monitoring of symptom recurrence could provide the temporal resolution needed to detect indicators of future depressive episodes [4]. Therefore, the use of RMTs in MDD could hold great potential for understanding current and predicting future depressive states.

### Remote Assessment of Disease and Relapse in MDD

Remote Assessment of Disease and Relapse in MDD (RADAR-MDD) is a longitudinal, multi-site, prospective cohort study that is investigating the feasibility and predictive validity of RMT data in identifying predictors of MDD relapse [2]. It is part of the wider RADAR-CNS program [12] and uses the open-source mHealth platform, RADAR-base [13], to collect aRMT data (fortnightly tracking of mood, self-esteem, and speech using an active smartphone app), pRMT data (GPS, Bluetooth interactions, and ambient noise and light using a passive smartphone app and heart rate and step count from a wrist-worn wearable), and 3-monthly outcome assessments (web-based) in participants with MDD. The core research team provided the initial enrollment session and support throughout the 2-year remote follow-up period. Data were collected from 623 participants across the London, Amsterdam, and Barcelona sites, and the study was concluded in April 2021. The results will explore whether multi-parametric RMTs can feasibly provide clinically relevant information and, if so, pave the way for translation of the platform into routine clinical practice and self-management of MDD.

### Engagement With RMTs in Research

The promise of research such as RADAR-MDD depends heavily on user engagement. Engagement with mHealth technologies can be defined as (1) a behavioral construct measured by objective completion statistics and (2) an experiential construct measured by focused attention and interest when interacting with the technology [14]. Qualitative studies suggest that service users endorse the use of RMTs in mental health care [15,16]. Successful recruitment into the RADAR-MDD study also suggests widespread interest in using remote symptom tracking for research [17]. However, past studies have reported varying rates of behavioral engagement during follow-up. Studies using app-based symptom tracking in cohorts with depression have reported low rates of data completion [18,19]. A wider review of RMT for health management found large variations in aRMT and pRMT use times [20]. Preliminary data from RADAR-MDD indicate that participants completed a median of 21 (IQR 9-31) out of a possible 52 aRMT questionnaires, and 52.3% (326/623) provided wearable data for over 75% of the participating days [21]. Iterative work on the RADAR-base platform has also addressed the challenges of deciphering between low user engagement and technical issues with the technology [22].

Behavioral engagement with RMTs in research is vital in reducing data missingness and bias and enhancing quality [23,24]. However, an understanding of experiential engagement with RMTs and the act of symptom tracking itself could prove of equal benefit for data completeness and long-term adherence. In a study using multi-parametric RMTs in bipolar disorder, experiential engagement measures (self-awareness of emotional health and learning about symptoms) positively correlated with increased behavioral engagement with symptom tracking using a smartphone app and Fitbit [25]. A holistic approach to measuring engagement is necessary for understanding the current lack of and promoting future engagement with RMT studies.

Several methods are available to promote engagement within the RMTs themselves. In addition to the presence of a contactable research team, which has been previously associated with increased engagement [17,24], in-app components work remotely within the technology. Push notifications are prompts that appear on the smartphone screen and can vary according to content and timing [26]. Following the Fogg behavioral model [27], notifications provide a trigger to perform a behavior, such as completing tasks on a manual food logging app [28]. Adding theoretically informed notification content, such as insights or tips for using self-monitoring, can further motivate the completion of mood scales [29]. The effects of notification frequency on engagement show mixed results [26,30,31]. Data visualization is a common technique used in mood monitoring apps [32]. Visually displaying data completion allows users to revisit progress and may prompt the *action* of continued data input [33]. This might be especially effective given that anticipatory pleasure is thought to predict motivation for reward in individuals with depression [34]. It is unclear which combination of in-app features can promote behavioral and experiential engagement with a multi-parametric symptom-tracking app in depression. Findings in this field would provide scalable solutions for engagement in RMT studies, higher quality results, and applications for implementation in clinical practice.

### Study Aims and Objectives

This study aims to test the effect of in-app components in a multi-parametric RMT platform on engagement with active and passive symptom tracking in MDD. A 2-armed randomized controlled trial will be used to compare the RADAR-base active app with an adapted app with insightful notifications and progress visualization aimed at promoting behavioral and experiential engagement. Engagement will be measured as provision of symptom tracking data collected through RMT over the 12-week study period and the degree to which participants feel experientially engaged with symptom tracking via the platform. It is hypothesized that participants using the adapted app will be better engaged in monitoring their symptoms, as measured by both behavioral engagement (completion of mood questionnaires) and experiential engagement (measures of attention, aesthetic appeal, and self-awareness). Process evaluation measures will also reveal participant experience with the engagement strategies used.

## Methods

### Ethics Committee Approval

This study was approved by the Psychiatry, Nursing and Midwifery Research Ethics Subcommittee at King’s College London (reference number: RESCM-20/21-21083) and registered as a clinical trial (reference number: NCT04972474).

### Study Design

**Figure 1:**
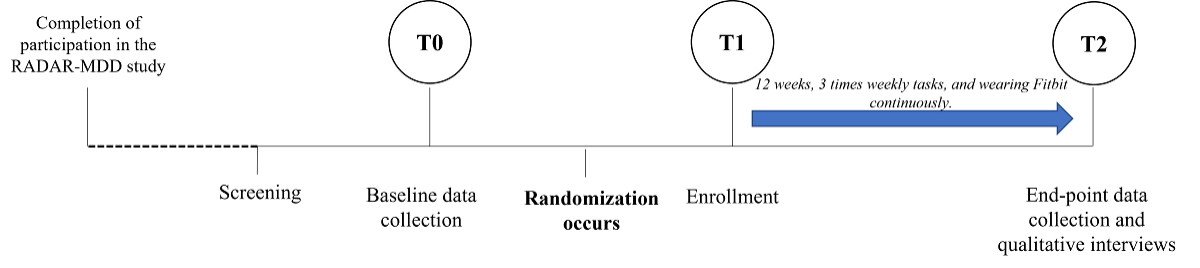
Study design from screening to follow-up end point, including time points 0, 1, and 2. RADAR-MDD: Remote Assessment of Disease and Relapse in Major Depressive Disorder.

This study is a 12-week, 2-arm randomized controlled trial with 1:1 randomization. A summary of the study design is presented in Figure 1. A 12-week period was chosen to align with the original structure of the RADAR-MDD study [2]. Participants will be recruited from RADAR-MDD, will provide baseline data (T0), and will be randomized at enrollment (T1) to 1 of 2 arms: an adapted app that includes insightful notifications and progress visualization (active arm) or active app as usual (control arm). Both the control and active arms will be delivered through the RADAR-base active app, which collects data in combination with a passive data collection app and a Fitbit Charge device [13]. In both arms, participants will be asked to complete 3 tasks each week via the app and wear the Fitbit device throughout the study. The primary outcome is the percentage of weekly tasks completed over 12 weeks of follow-up.

Upon completion of the study, 20 participants (n=10 from each arm) will also be randomly invited to complete a qualitative interview about their experience of participating.

The study will be conducted using a combination of the RADAR-base platform, including the management portal web application [13] and REDCap (Research Electronic Data Capture) [35]. Owing to the COVID-19 pandemic, participation will be fully remote.

### Eligibility Criteria

#### Inclusion Criteria

Participants will be included if they (1) participated in RADAR-MDD and gave consent for future research contact, (2) experienced at least one episode of MDD in the 2 years preceding RADAR-MDD enrollment, (3) are willing and able to continue to use an Android smartphone and Fitbit Charge device for a 12-week period (both provided for use in RADAR-MDD), and (4) feel comfortable completing an enrollment session remotely either via email instructions or video calls.

#### Exclusion Criteria

Participants will be excluded if they have been diagnosed with a comorbid psychiatric disorder since their enrollment into RADAR-MDD: bipolar disorder, schizophrenia, psychosis, schizoaffective disorder, or dementia. This will be checked with the participant via email during the recruitment process.

### Recruitment

Participants will be recruited through the RADAR-MDD database of the London site. Contact details will be extracted from the RADAR-MDD REDCap system for those who have provided consent to be contacted for future research. This will include any participant who enrolled in the study during the 31 months of recruitment (November 2017 to June 2020; n=345).

Participants will be invited to participate via an email that will explain the study and provide the participant information sheet. Interested participants will respond via email. They will then be asked the eligibility questions via email. If eligible, participant details will be entered into the study REDCap system, which will initiate the sending of a personalized link to the web-based consent form and baseline questionnaires (T0). Once these have been completed, the participant will receive a second link to a web-based booking system, where they can book a time slot for an enrollment session. Enrollments will be conducted between April and May 2021. On the day of enrollment, participants will receive an email (or a video call, depending on their preference) outlining instructions for downloading the study apps and unique QR codes to register them to the platform (T1).

### Interventions

#### Overview

Upon enrollment into the study, participants will be asked to complete 3 tasks per week via the active app, allow the passive app to run in the background on their smartphones, and wear the Fitbit device as much as possible. The active app tasks are as follows: (1) Patient Health Questionnaire-8 (PHQ-8) [36], an 8-item questionnaire assessing the variability of depressive symptoms over the last week; (2) Rosenberg Self-Esteem Scale [37], a 10-item questionnaire assessing variations in self-esteem; and (3) a speech task, during which the participant records themselves reading aloud a short paragraph.

In both arms, the weekly questionnaire tasks, the passive app, and the Fitbit device remain the same. The study is designed such that the enrollment process is identical for both arms to ensure that participants do not prime to the study arm that they are assigned and that both arms are comparable with RADAR-MDD.

#### Control Condition

Participants in the control arm will receive one notification at the 9 AM, 10 AM, and 11:30 AM time points on the day that a questionnaire task is due, which reads “Questionnaire Time. Won’t usually take longer than 3 minutes*.*” They will not be able to view any data aside from that through the Fitbit app.

#### Active Condition

##### Development of the Adapted App

The design of RADAR-MDD, including the active app, was heavily informed by service user involvement [38]. This study used behavior change theory and further patient public involvement work to inform its design.

**Figure 2:**
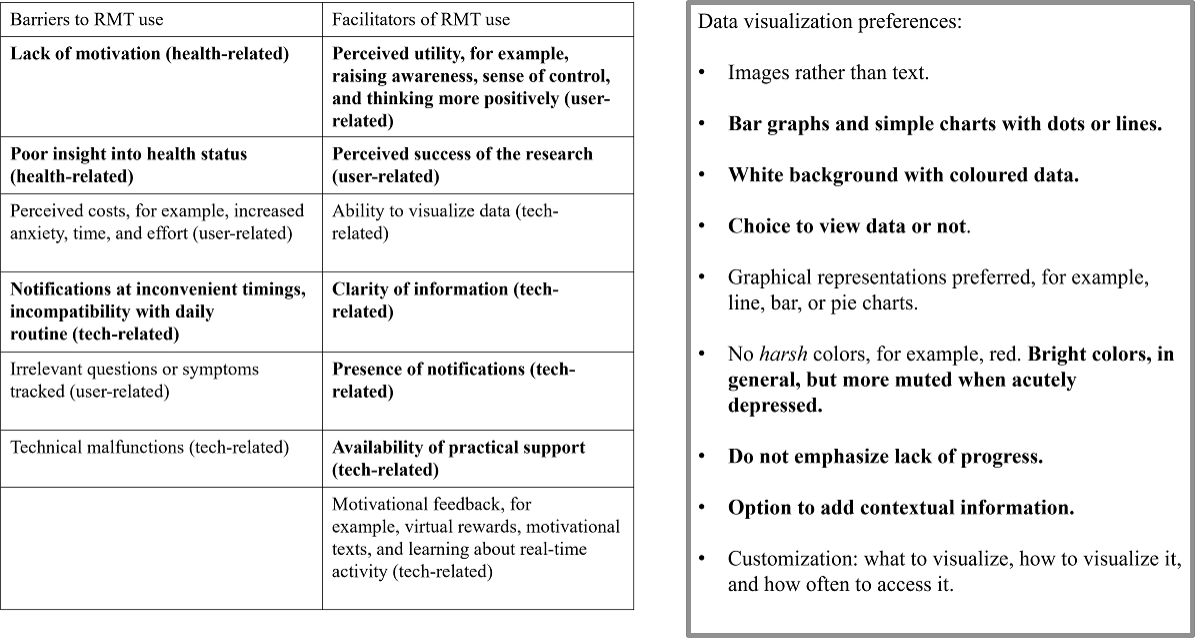
Service user involvement in the design of the adapted app. RMT: remote measurement technology.

To establish how best to promote the behavior of symptom tracking, it is useful to draw on theories and models of behavior change. The Behavior Change Wheel [39] presents a framework for the development of strategies to promote a target behavior. Previous work was used to identify key health-, user-, and technology-related barriers to engaging with symptom tracking in MDD [15,20,38] (Figure 2). Following the COM-B model, *psychological capabilities*, such as lack of symptom insight and perceived utility of the research, *automatic motivations* related to motivational difficulties and low mood, and *physical opportunities*, such as inability to answer questionnaires at a specific time and unsure if the data have been logged, presented the most pertinent barriers. Following the Behavior Change Wheel, suitable intervention functions thus included education, incentivization, and enablement [39]. Therefore, it was decided that an engaging app should include reminder notifications with information on the potential impacts of symptom tracking from a credible source. It should also include incentivizing feedback on behavior in the form of data visualization. Finally, users should be provided with researcher contact details to report technical issues or receive support.

The progress visualization component was further informed by service user involvement [40] (Figure 2). Simple, clear graphical representations of data were preferred, presented on a white background with colored data points. Users expressed an interest in positive reinforcement based on reaching achievements, for example, step count goals or simply the entering of data, coupled with a visual representation of completion, for example, a change in color. They also requested the choice to view or hide visualizations. Therefore, the visualization component was designed to comprise a separate section of the app that users can choose to view with a simple, colored graph showing completion or noncompletion at each weekly time point. Completion is denoted by a green dot and noncompletion, by a red dot.

##### In-App Components

**Figure 3:**
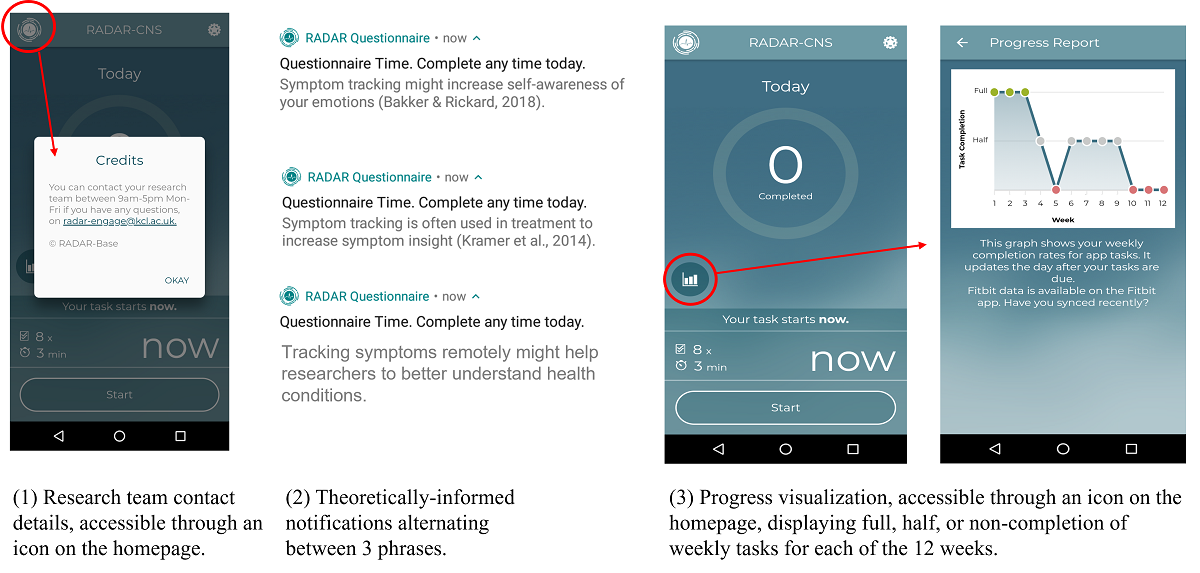
Screenshots of the in-app components included in the adapted app.

Participants in the active arm will receive notifications at the same time points as those in the control arm, along with the following additional content (Figure 3):

1. Theoretically informed notifications: additional sentences included in the notifications covering the proposed benefits of remote symptom monitoring for emotional self-awareness, clinical practice, and research, along with a reminder that the questionnaire can be completed *any time today.*
2. Progress visualization: participants will be able to view their questionnaire task completion through the app visualized as a graph that is accessible from the main app home page.
3. Research team contact details: additional text on the app home page will provide a contact phone number, email address, and contact times for the research team.

### Data Collection and Follow-up Procedure

A summary of measures and data collection time points is outlined in Table 1.

**Table 1:** A summary of measures and data collection points across the 12-week follow-up period.

Measures		Baseline (T0)	End point (T2)	Weekly	Continuously
**REDCap^a^ survey**					
	Consent	✓			
	Contact information	✓			
	Study devices	✓			
	Sociodemographics	✓			
	Social environment	✓			
	Medical history	✓			
	Lifetime Depression Assessment Self-report [41]	✓			
	Inventory of Depressive Symptomatology [42]	✓	✓		
	The World Health Organization Composite International Diagnostic Interview Short-Form [43]	✓	✓		
	Generalized Anxiety Disorder-7 [44]	✓	✓		
	Work and Social Adjustment Scale [45]	✓	✓		
	Brief Illness Perception Questionnaire [46]	✓	✓		
	Life events [47]	✓	✓		
	Client Service Receipt Inventory [48]	✓	✓		
	User Engagement Scale (adapted for mHealth^b^ use) [49]	✓	✓		
	Emotional Self-Awareness Questionnaire [50]	✓	✓		
	mHealth App Usability Questionnaire [51]	✓	✓		
**Active app measures**					
	Patient Health Questionnaire-8 [36]			✓	
	Rosenberg Self-Esteem Scale [37]			✓	
	Speech task			✓	
**Passive app measures**					
	GPS, Bluetooth, and ambient noise and light				✓
**Fitbit**					
	Heart rate and step count				✓
**Process evaluation**					
	App use metrics				✓
	Qualitative interviews		✓^c^		

^a^REDCap: Research Electronic Data Capture.

^b^mHealth: mobile health.

^c^Only a select few participants will be asked to interview.

Baseline questionnaires will comprise questions on contact information, sociodemographics, recent service use, physical and mental health history, and comorbidities, including the presence of depression and recent life events. The research team will also manually pull data pertaining to participation in RADAR-MDD for each participant, for example, participation length and completion rates. At the 12-week postbaseline follow-up, participants will receive a personalized link to repeat several baseline questionnaires. Responses received more than 3 weeks after baseline or follow-up will not be recorded.

The principal investigator (KMW) will monitor incoming data streams to ensure that the app is functioning correctly. Participants will not be contacted by the team once enrollment is complete, aside from a check-in email at the 6-week point to ensure that the app is functioning correctly. Participants will not be withdrawn from the study based on nonengagement; however, participants will be made aware that they can withdraw at any point.

Suicidal ideation will also be monitored at baseline (T0) and at follow-up (T2). Participants who report ideation or intent at either time point will be contacted via phone call, advised to contact their treating physician, and emailed a list of appropriate signposts.

Upon completion of the study, participants will view a debriefing page explaining that the study aimed to test the effectiveness of notifications and progress visualizations on engagement with the platform. Both arms will be outlined, identifying arm assignments and end point instructions.

### Outcome Measures

#### Primary Outcome Measure

The primary outcome measure will be the behavioral engagement with the RADAR-base system. This will be measured as the percentage of weekly PHQ-8 questionnaires completed over the 12-week follow-up period. Completion of 1 PHQ-8 task is defined as the completion of the 8 questions.

#### Secondary Outcome Measures

Secondary outcome measures will be as follows:

1. Experiential engagement with the RADAR-base platform measured with the User Engagement Scale (UES) [52] adapted to mHealth use [49]. The UES is a 30-item questionnaire measuring 4 factors of experiential engagement with mHealth apps: focused attention, perceived usability, aesthetic appeal, and reward. The UES has been widely adopted and shows good reliability and construct validity [53].
2. Experiential engagement with the RADAR-base platform measured by the Emotional Self-Awareness Questionnaire (ESQ) [50]. The ESQ is a 33-item questionnaire measuring recognition, contextualization, and decision-making in relation to one’s own emotions. The ESQ has a reliability of 0.92 and shows significant positive correlations with the Emotional Intelligence Test [50].
3. System usability measured using the mHealth App Usability Questionnaire (MAUQ) for stand-alone apps used by patients [51]. This will be assessed at T2 only, asking participants to reflect solely on their experiences over the last 12 weeks. The MAUQ comprises 18 questions relating to the immediate and long-term usability of the app, including health care management (overall Cronbach α=.914).
4. Combined adherence to active and passive (Fitbit) components of the system will be measured as follows: adherence rate for the active app measured as the proportion of participants with over 50% of completed data across all 3 weekly questionnaire tasks and adherence rate for the passive Fitbit measured as the proportion of participants with over 50% of study days with any recorded data. These measures were chosen to align with data availability reporting in RADAR-MDD [21], previous literature [25], and the minimum amount of data sufficient for performing predictive analyses.

#### Additional Data Collection

Passive data through the RADAR-base passive app will also be collected; however, these will not be analyzed as part of this trial. This additional data will be collected for 2 reasons: (1) to emulate the RADAR-MDD as closely as possible and (2) for use in future analyses. The passive app collects information on phone location, battery level, Bluetooth devices, and background noise and light. Participants can opt out of using any of the study apps during their participation.

#### Process Evaluation Measures

Process evaluation measures will also be collected to further understand the interaction with the RADAR-base system. Quantitative measures will be obtained regarding app use: notification interaction, app initialization, specific module viewing, and viewing time. These will be available from the back end of the RADAR-base platform.

At the end of the follow-up period, 20 participants will be randomly invited to participate in a semistructured telephone interview with a member of the research team, discussing their experiences of participating in the study. Discussions will comprise perceptions of the arm to which the participant was randomized, experiences of the in-app techniques used, suggestions for further improvements for engagement with the system, and views on engagement with RMT systems for symptom tracking in research, clinical care, and self-management (Multimedia Appendix 1).

### Sample Size

Power calculations were performed based on preliminary data from the RADAR-MDD. A total of 132 participants are required to detect a difference of 25% completion of PHQ-8 tasks between the control and active arms, with 80% power and 95% CI at the 12-week end point. Allowing for 10% attrition (based on previous research [21] but accounting for a much shorter follow-up period in this study), we will aim to recruit 140 participants. A total of 345 participants will be available to be contacted from the RADAR-MDD study cohort; assuming 50% acceptance of invitation (given the recruitment from a previously motivated cohort), a target of 140 participants should be feasible.

### Randomization

Randomization will occur after baseline data collection when the REDCap randomization module initiates the generation of a QR code from the RADAR-base management portal assigned to the participant identifier. Each participant will be randomly allocated at a 1:1 ratio to either the control or the active arm. Simple randomization will be used, in which an allocation table with a random sequence of 1,2 will be generated and uploaded to REDCap. This will be carried out by a team member external to the core research team (YR) and therefore be concealed from the principal investigator (KMW) before enrollment.

### Blinding

Individual participants will have previously used the RADAR-MDD app and therefore cannot be blinded because they might recognize new features of the app. However, arm assignments will not be explicitly revealed to the participants until the study debrief.

The principal investigator (KMW) will be unblinded to allocation to ensure that remote enrollments have been carried out correctly. All measures are conducted using the app or web-based REDCap system to avoid detection bias in assessments [54]. The trial data manager (DL) will be blind to arm allocation. No other individuals will have access to the data set for data monitoring or analysis purposes; all tasks will be carried out by the principal investigator (KMW).

### Data Management

All data collected via the Fitbit device and smartphone apps will be encrypted and uploaded to a secure server maintained by King’s College London in accordance with the process cited by Ranjan et al [13]. The REDCap system sits on the King’s College London Rosalind server. Only members of the RADAR-Engage team will have access to identifiable data. Qualitative interview data will be temporarily stored on the King’s College London server, transcribed anonymously, and subsequently deleted.

### Statistical Analysis: Plan

#### Overview

All data, including those from withdrawn participants (unless they request for their data to be deleted) will be included in the final analysis. Demographic and clinical characteristics at baseline and follow-up will be summarized by arm using appropriate summary statistics, for example, mean and SD for continuous variables and counts and percentages for categorical variables. Data completeness for all measures and outcomes will be summarized.

The primary outcome will be analyzed using 2-sample *t* tests (2-tailed) to assess whether the mean percentage of PHQ-8 completion in each arm is statistically different.

For the secondary outcomes, experiential engagement (as measured by the UES and ESQ) will be collected at T0 and T2 and will thus be calculated as a change from baseline. This will be assessed using repeated measures mixed modeling to explore whether experiential engagement is statistically different between the 2 arms. App usability scores (MAUQ) and overall system adherence rates will also be compared. Complete case analyses will be used; if <20% of responses to each questionnaire are missing, mean imputation will be used to provide a total score.

All analyses will be conducted using the intention-to-treat principle. The threshold for statistical significance was set at *P*=.05.

#### Process Evaluation Analyses

Qualitative interviews will be transcribed and coded using NVivo software [55]. Grounded theory thematic analysis will provide an exploration of participant experiences across the 2 arms and with the additional in-app components. Descriptive statistics will be reported for app use statistics.

### Dissemination

This study will be reported following the CONSORT (Consolidated Standards of Reporting Trials) checklist [56]. The results of this study will be discussed via publication.

## Results

This study will begin recruiting and enrolling participants in April and May 2021. Data collection will be completed by September 2021. Data analysis will commence in 2022. The results of this study will be communicated via publication in mid-2022.

## Discussion

### Principal Findings

The use of RMTs for symptom tracking in MDD research holds great potential for relapse prediction and personalized health care. Understanding current and promoting future engagement with RMTs in research studies is of utmost importance for producing high-quality results, and this is only amplified by the shift to remote health care monitoring during the COVID-19 pandemic [57-59]. Although previous studies have explored the impact of specific in-app components in encouraging data completion [25,26,32,33], to our knowledge, this study is one of the first to explore the promotion of engagement with a multi-parametric RMT system for MDD symptom tracking. Within the framework of the RADAR-base system, this study uses the questionnaire app as a participant-facing conduit to promote behavioral and experiential engagement with active and passive RMT in a large-scale research study incorporating theoretical notifications and progress visualization.

The findings of this study will, first, provide some understanding about how best to promote engagement in subsequent rounds of the RADAR-MDD study. The ability to collect sufficient data remotely by relying less heavily on a core research team while also minimizing burden on the user is a highly valuable asset for RMT research. This study also represents the first attempt to recruit and follow up with participants completely remotely using RADAR-base and, if successful, will pave the way for fully remote recruitment across a range of conditions. Second, this work sheds light on experiential engagement with RMT symptom tracking. The findings here could uncover new methods for measuring and promoting engagement in MDD research. Third, studying behavioral and experiential engagement in a research context can act as a proxy for understanding engagement in a clinical context [60]. Taken together, these findings could have wider implications for RMT research studies across health conditions, alongside the implementation of RMT data collection in clinical settings.

### Strengths and Limitations

A key strength of this study is its grounding in a previous research project, using a system that has already been well-documented, designed, and developed for the purpose of RMT data collection [21,61,62]. It also takes an additional theory-driven and user-centered approach to adapting components of the system to promote optimal user engagement. However, this study has 3 main limitations. First, our ability to recruit and retain a sufficient number of participants for power analysis may be hindered by participation fatigue, given that many will have completed up to 2 years in the previous study. The effects of the COVID-19 pandemic on participants’ willingness to engage in research studies are unclear. Second, it should be considered that recruiting from an existing study cohort with prior understanding of the system could create a ceiling effect for engagement, such that participants are already highly motivated to engage in symptom tracking. App literacy has also been noted as a key facilitator of mHealth app engagement [63]. Nonetheless, there is good reason to believe that the new in-app components can encourage engagement over and above the moderate data availability reported in RADAR-MDD [21]. Third, although concerted efforts were made to include health-, user-, and technology-related barriers to engagement in the app development process, we acknowledge that this is not all-encompassing. Certain aspects of depressive symptomatology, for example, low mood or motivation [34], could affect engagement with the RADAR-base system in ways that might not be mitigated by theoretical notifications or progress visualization. Therefore, we have also included process evaluation measures to further understand how participants interact with the components and gain insight for future improvements.

## Appendix

Multimedia Appendix 1 Semistructured qualitative interview schedule (N=20).

## Abbreviations

aRMT: active remote measurement technology
CONSORT: Consolidated Standards of Reporting Trials
ESQ: Emotional Self-Awareness Questionnaire
MAUQ: mHealth App Usability Questionnaire
MDD: major depressive disorder
mHealth: mobile health
PHQ-8: Patient Health Questionnaire-8
pRMT: passive remote measurement technology
RADAR-MDD: Remote Assessment of Disease and Relapse in Major Depressive Disorder
REDCap: Research Electronic Data Capture
RMT: remote measurement technology
UES: User Engagement Scale
